# High-Fructose/High-Fat Diet Downregulates the Hepatic Mitochondrial Oxidative Phosphorylation Pathway in Mice Compared with High-Fat Diet Alone

**DOI:** 10.3390/cells11213425

**Published:** 2022-10-29

**Authors:** Milton D. Chiang Morales, Chao-Yuan Chang, Van Long Le, I-Tao Huang, I-Lin Tsai, Hung-Jen Shih, Chun-Jen Huang

**Affiliations:** 1 International Ph.D. Program in Medicine, College of Medicine, Taipei Medical University, Taipei 110, Taiwan; 2 Graduate Institute of Clinical Medicine, College of Medicine, Taipei Medical University, Taipei 110, Taiwan; 3 Department of Medical Research, Wan Fang Hospital, Taipei Medical University, Taipei 116, Taiwan; 4 Integrative Research Center for Critical Care, Wan Fang Hospital, Taipei Medical University, Taipei 116, Taiwan; 5 Department of Anesthesiology and Critical Care, Hue University of Medicine and Pharmacy, Hue City 52000, Vietnam; 6 Emergency Department, Redcliffe Hospital, Redcliffe, QLD 4020, Australia; 7 School of Public Health, Faculty of Medicine, University of Queensland, Herston, QLD 4006, Australia; 8 Department of Biochemistry and Molecular Cell Biology, School of Medicine, College of Medicine, Taipei Medical University, Taipei 110, Taiwan; 9 Division of Urology, Department of Surgery, Changhua Christian Hospital, Changhua 500, Taiwan; 10 Department of Urology, School of Medicine, College of Medicine, Taipei Medical University, Taipei 110, Taiwan; 11 Department of Anesthesiology, Wan Fang Hospital, Taipei Medical University, Taipei 116, Taiwan; 12 Department of Anesthesiology, School of Medicine, College of Medicine, Taipei Medical University, Taipei 110, Taiwan

**Keywords:** liver, mitochondria, high-fat diet, high fructose, proteomic, OXPHOS

## Abstract

Both high-fat diet (HFD) alone and high-fructose plus HFD (HFr/HFD) cause diet-induced non-alcoholic fatty liver disease in murine models. However, the mechanisms underlying their impacts on inducing different levels of liver injury are yet to be elucidated. This study employed a proteomic approach to elucidate further on this issue. Adult male C57BL/6J mice were allocated to the HFD or the HFr/HFD group. After feeding for 12 weeks, all mice were euthanized and samples were collected. The proteomic profiles in liver tissues were analyzed using liquid chromatography–tandem mass spectrometry followed by canonical pathway analysis. We demonstrated that the mitochondrial oxidative phosphorylation (OXPHOS) pathway was the most significantly downregulated canonical pathway in the HFr/HFD group when compared with the HFD group. Within the OXPHOS pathway, the HFr/HFD group demonstrated significant downregulation of complexes I and III and significant upregulation of complex IV when compared with the HFD group. Moreover, the HFr/HFD group had lower protein levels of NADH: ubiquinone oxidoreductase subunits S3, S6, A5, and A12 in complex I (*p* < 0.001, =0.03, <0.001, and <0.001, respectively), lower protein level of cytochrome C in complex III (*p* < 0.001), and higher protein level of cytochrome C oxidase subunit 2 in complex IV (*p* = 0.002), when compared with the HFD group. To summarize, we have demonstrated that the hepatic mitochondrial OXPHOS pathway is significantly downregulated in long-term HFr/HFD feeding when compared with long-term HFD feeding. These data support the concept that the hepatic mitochondrial OXPHOS pathway should be involved in mediating the effects of HFr/HFD on inducing more severe liver injury than HFD alone.

## 1. Introduction

Obesity is a major risk factor for the development of non-alcoholic fatty liver disease (NAFLD), amongst numerous other chronic liver diseases [1]. NAFLD is the most common liver disease at approximately 32% prevalence worldwide [2]. NAFLD is closely associated with metabolic derangements and is considered as a hepatic manifestation of metabolic syndrome [3,4]. Notably, there is a wide spectrum of liver pathologies caused by NAFLD, ranging from simple steatosis to advanced fibrosis, cirrhosis, and even hepatocellular carcinoma [5].

Diets containing a high saturated fat and fructose content are associated with metabolic syndrome, obesity, coronary heart disease, and type 2 diabetes [6,7]. Animal (e.g., murine) diet-induced obesity models have thus been developed, achieved via long-term feeding of high-fat diet (HFD) and high-fructose plus HFD (HFr/HFD) [1,8]. Both HFD and HFr/HFD models have also been shown to induce NAFLD [9]. Interestingly, existing literature suggests that HFr/HFD may induce more severe liver injury than HFD alone [5]. García-Berumen et al. reported that rats fed with HFD exhibited an intermediate-degree liver injury and approximately 40% hepatocytes demonstrated microvesicular steatosis; rats fed with HFr/HFD exhibited more severe liver injury and approximately 60% hepatocytes demonstrated microvesicular steatosis [10]. As such, HFr/HFD is more widely utilized in the study of NAFLD compared with HFD [11].

Although both HFr/HFD and HFD models induce NAFLD, the molecular mechanism(s) underlying their different effects in inducing liver injury remains to be elucidated. Proteomics, a powerful tool for studying (mal)adaptation across multiple tissues and biological fluids in pathophysiological conditions, may be a useful technique for this investigation [12]. The liver is a suitable tissue for proteomic analysis given the abundance of secreted proteins [12]. The detection of protein expression patterns using proteomic analysis has been demonstrated in previous studies on the impact of HFD on mouse liver [13,14,15,16].

We subjected murine HFr/HFD and HFD models to proteomic analysis to investigate their underlying mechanisms of liver injury. A cluster of proteins that showed changes associated with these two models and the relevant pathways were identified using high-throughput proteomic tools, liquid chromatography-tandem mass spectrometry (LC-MS/MS), followed by canonical pathway analysis. The specific elements in the identified pathways were then analyzed for validation.

## 2. Materials and Methods

### 2.1. Animals and Approval of Studies

All studies involving animals were approved by the Institutional Animal Use and Care Committee of Taipei Medical University (LAC-2017-0454). A group of 7-week-old male C57BL/6J mice (National Laboratories Animal Center, Taipei, Taiwan) was used in this study. The mice were fed a standard laboratory diet (Laboratory Lab Diet 5001, Lab Diet, St. Louis, MO, USA), had unlimited access to water, and were kept in the light for 12 h and in the dark for 12 h. The mice were treated and maintained according to the US National Institutes of Health guidelines.

### 2.2. Animal Model of HFD and HFr/HFD

The mice were acclimatized to laboratory conditions for 1 week before the study and then randomized into the HFD or the HFr/HFD group. The HFD group was fed with 60% kcal from fat, 20% kcal from carbohydrate, and 20% kcal from protein (Research Diets, New Brunswick, NJ, USA) for 12 weeks, consistent with prior protocols [17]. As an experimental group for the NAFLD model, another group of mice was fed a HFD, as above-mentioned, plus 30% high-fructose (D-(-)-Fructose sc-221456B, Santa Cruz Biotechnology, Inc., Dallas, TX, USA) in their drinking water for 12 weeks, also consistent with prior protocols [18,19,20].

### 2.3. Harvesting and Analysis of Blood Samples

After overnight fasting, mice were anesthetized with intraperitoneal injection of zoletil/xylazine (40/10 mg/kg). All mice then underwent a midline laparotomy. An aortic puncture was performed to collect blood samples. Blood samples underwent centrifugation at 7000 rpm and 4 °C for 15 min (Eppendorf 5415R Refrigerated Centrifuge, Sigma-Aldrich, Burlington, MA, USA) and the plasma was collected and stored at −80 °C. Plasma concentrations of glucose, triglyceride, total cholesterol, low-density lipoprotein (LDL), and high-density lipoprotein (HDL) levels were measured for metabolic profile assessment (Roche Hitachi 917 Chemistry Analyzer; International Diagnostic Equipment, LLC, Temecula, CA, USA). Moreover, plasma concentration of very low-density lipoprotein (VLDL) was measured using a mouse sandwich enzyme-linked immunosorbent assay kit (ELK Biotechnology, Wuhan, China), and performed as per the manufacturer’s protocol.

### 2.4. Harvesting Liver Tissues and Histological Analysis

After euthanasia by decapitation, liver tissues were dissected and harvested. Part of the liver tissues were snap frozen and then stored at −80 °C for later analysis. Part of the liver tissues were fixed in 10% formalin (Sigma-Aldrich) and embedded in paraffin wax for histological analysis. After serial sectioning, hematoxylin and eosin staining was performed. Liver tissues were assessed at ×200 magnification under light microscopy (Olympus FV 100, Olympus, Tokyo, Japan) for morphological characteristics indicating degrees of liver injury and NAFLD progression. Characteristics included vacuolation and ballooning degeneration, focal necrosis, congestion and hemorrhage, steatosis, polymorphonuclear neutrophil infiltration and lobular inflammation. By calculating Suzuki scores (namely, sums of vacuolation [0: none; 4: severe], necrosis [0: none; 4: >60%], and congestion [0: none; 4: severe]), an assessment of liver injury degree was performed [21]. This study also employed the NAFLD activity score system to assess the degree of NAFLD progression [22]. By calculating a NAFLD activity score (namely sums of steatosis [0: none; 3: severe], inflammation [0: none; 3: >4 foci], and ballooning degeneration [0: none; 2: many ballooned cells]), an assessment of NAFLD progression degree was performed [22].

### 2.5. LC-MS/MS

Mouse liver samples were prepared as previously performed for mass spectrometry [23]. Briefly, the diluted protein solutions of liver tissues were digested at 37 °C with sequencing-grade modified porcine trypsin (Promega, Madison, WI, USA). To extract peptides, the solutions were desalted, centrifuged, and stored at −80 °C. The digested peptides were eluted on a preparative reversed-phase column (Zorbax 300SB-C18, 0.3 × 5 mm^2^; Agilent Technologies, Wilmington, DE, USA), then eluted on a custom column (HydroRP 2.5 µm, 75 µm I.D. × 20 cm. with a 15 µm peak) with high-performance liquid chromatography buffer B (99.9% acetonitrile/0.1% formic acid) for 70 min. The liquid chromatography apparatus (Dionex RSLC system; Thermo Fisher Scientific, San Jose, CA, USA) with electrospray interface was coupled with the Orbitrap Elite™ Hybrid Ion Trap-Orbitrap Mass Spectrometer (Thermo Fisher Scientific) operated with Xcalibur 2.2 software (Thermo Fisher Scientific). Mass spectrometry was performed in data-dependent acquisition mode with internal calibration, followed by 1 full MS scan and 20 MS/MS scan events for the 20 most abundant precursor ions in full MS scan. Automatic amplification control was set to a maximum accumulation time of 100 ms or 3 × 10^6^ ions for full MS scans and 200 ms or 3 × 10^3^ ions for MS/MS scans.

### 2.6. Protein Identification and Quantification

To identify and quantify proteins, the Proteome Discoverer software (version 2.2; Thermo Fisher Scientific) was used. MS/MS spectra were compared with Swiss-Prot data (version 2.5; Matrix Science, London, UK). Precursor and fragment ion mass tolerances were set at 10 ppm and 0.5 Da, respectively for peptide identification. A maximum of two missed cleavages were permitted for tryptic peptides. Only aligned peptide spectral matches with high confidence (*p* < 0.05) were filtered for reliable peptide identification with an overall false discovery rate < 0.01. Proteins with a single peptide hit were removed. Spectral counting was used for label-free semi quantitation of protein levels in liver tissues to estimate comparative protein abundance [24]. We calculated the normalized spectral counts of each protein by dividing its spectral counts by the total spectral counts of the samples and multiplying that average total spectral count by the protein spectra. The fold change of the normalized spectral coefficients for each protein in the HFr/HFD group relative to the HFD group was recorded.

### 2.7. Functional Enrichment Analysis Using Ingenuity Pathway Analysis (IPA)

Functional enrichment analysis was conducted with proteins that were differentially expressed in HFr/HFD and HFD groups based on a fold change of over or under 1.5 using IPA (Qiagen Inc., Valencia, CA, USA). In both the HFr/HFD and the HFD groups, enriched canonical pathways were verified using Fisher’s exact tests, with the logarithm of the unadjusted *p*-values presented in log form. In this study, pathways with log(p) greater than 1.3 (*p* < 0.05) were considered significant. An activation state of canonical pathways was represented by a z-score.

### 2.8. Immunoblotting Assay

For validating protein(s) identified with IPA, an immunoblotting analysis was performed on liver tissues. Using a previously described method, protein extraction was carried out on freshly frozen liver tissues [25]. Equal amounts of proteins were separated by electrophoresis and transferred to nitrocellulose membranes (Bio-Rad Laboratories, Hercules, CA, USA). The membranes were then incubated overnight with either the primary antibody against the identified protein(s) or the internal standard Actin (anti-Actin, A5441, Sigma-Aldrich). Bound antibody was detected by chemiluminescence (ECL plus kit; Amersham BioSciences, Buckinghamshire, UK) and densitometric analysis was performed using a free image processing software (VisionWorks® Software, Version 8.20., Analytik Jena US, Inc., Upland, CA, USA; accessed on 1 July 2021) to measure band density.

### 2.9. Statistical Analysis

An analysis of variance followed by a post hoc pairwise comparison was performed using Tukey’s test to determine the between-group differences. Data were presented as the mean ± standard deviation. A *p* value of <0.5 was considered as significant. We performed the statistical analyses using the prism package in GraphPad (GraphPad Software, Inc., La Jolla, CA, USA).

## 3. Results

### 3.1. Effects of HFD and HFr/HFD Feeding on Inducing Obesity in Mice

**Figure 1:**
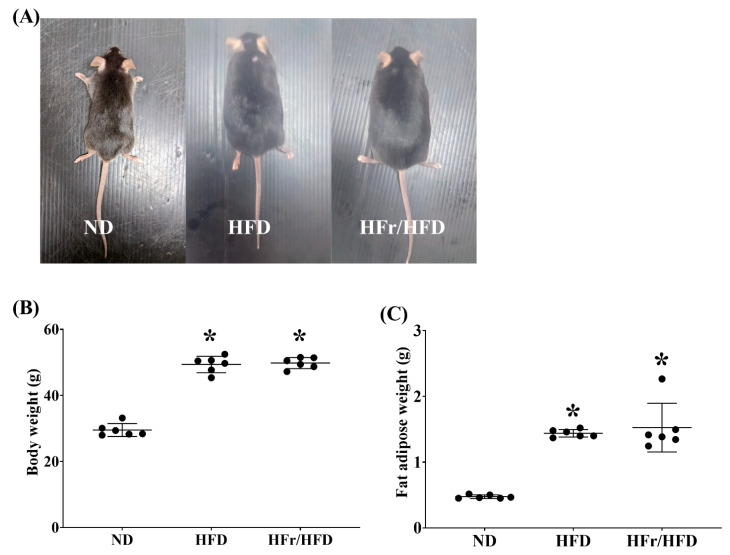
(**A**) Photographs of representative male C57BL/6J mice fed for 12 weeks with normal diet (ND), high-fat diet (HFD) and high-fructose/high-fat diet (HFr/HFD). (**B**) Comparison of mean body weight of the ND, HFD, and HFr/HFD groups. (**C**) Comparison of mean fat adipose weight of the ND, HFD, and HFr/HFD groups. Data were obtained from 6 mice in each group and presented as mean ± standard deviation. ND: normal diet group. HFD: the high-fat diet group. HFr/HFD: the high-fat diet plus 30% fructose in the drinking water group. * *p* < 0.05, versus the ND group.

Figure 1A illustrates the difference in body size in mice fed with normal diet (ND), HFD, and HFr/HFD (the ND, HFD, and HFr/HFD groups, respectively). After feeding for 12 weeks, the body weights in mice of the HFD and HFr/HFD groups were significantly higher than that in mice of the ND group (both *p* < 0.001; Figure 1B), whereas the body weights in mice of the HFD and HFr/HFD groups were not significantly different (*p* = 0.94; Figure 1B). Similarly, the fat adipose weight in mice of the HFD and HFr/HFD groups were both significantly higher than that in mice of the ND group (both *p* < 0.001; Figure 1C); the fat adipose weights in mice of the HFD and HFr/HFD groups were not significantly different, either (*p* = 0.77; Figure 1C). 

### 3.2. Metabolic Profile 

**Figure 2:**
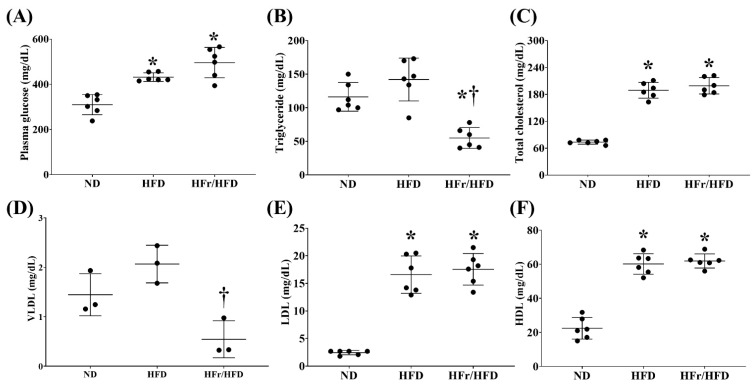
Plasma concentration of (**A**) glucose, (**B**) triglyceride, (**C**) total cholesterol, (**D**) very low-density lipoprotein (VLDL), (**E**) low-density lipoprotein (LDL), and (**F**) high-density lipoprotein (HDL). Glucose, triglyceride, total cholesterol, LDL, and HDL data were obtained from 6 mice in each group and VLDL data were obtained from 3 mice in each group. Data were presented as mean ± standard deviation. ND: normal diet group. HFD: high-fat diet group. HFr/HFD: high-fat diet plus 30% fructose in drinking water group. * *p* < 0.05, versus the ND group; † *p* < 0.05, the HFr/HFD group versus the HFD group.

Figure 2 demonstrates the metabolic profile data. Figure 2A illustrates the fasting plasma glucose levels of the ND, HFD, and HFr/HFD groups. Hyperglycemia was observed in mice of the HFD and HFr/HFD groups. The fasting plasma glucose concentrations in mice of the HFD and HFr/HFD groups were both significantly higher than that in mice of the ND group (*p* = 0.001 and <0.001, respectively). On the other hand, the fasting glucose levels in mice of the HFD group and HFr/HFD groups were not significantly different (*p* = 0.08).

Similar pictures were observed in the data of total cholesterol (Figure 2C), LDL (Figure 2E), and HDL (Figure 2F). In both the HFD and HFr/HFD groups the fasting plasma concentrations of total cholesterol, LDL, and HDL were all significantly higher than those of the ND group (all *p* < 0.001). Moreover, the fasting plasma concentrations of total cholesterol, LDL, and HDL in the HFD and HFr/HFD groups were not significantly different (*p* = 0.97, =0.91, and =0.85, respectively). 

Figure 2B,D illustrate the fasting plasma triglyceride and VLDL levels of the ND, HFD, and HFr/HFD groups. Notably, a trend of higher fasting plasma triglyceride and VLDL concentrations in the HFD group than in the ND group was observed. However, the difference in the fasting plasma triglyceride and VLDL concentrations between the HFD and ND group did not reach statistical significance (*p* = 0.18 and =0.13, respectively). In contrast, the fasting plasma triglyceride concentration in the HFr/HFD group was significantly lower than those in the ND and HFD groups (*p* = 0.001 and <0.001, respectively). The fasting plasma VLDL concentration in the HFr/HFD group was also significantly lower than that in the HFD group (*p* = 0.008). A trend of lower fasting plasma VLDL concentration in the HFr/HFD group than that in the ND group was also observed; whereas the between group difference did not reach statistical significance (*p* = 0.051).

### 3.3. Degrees of Liver Injury and NAFLD Progression

**Figure 3:**
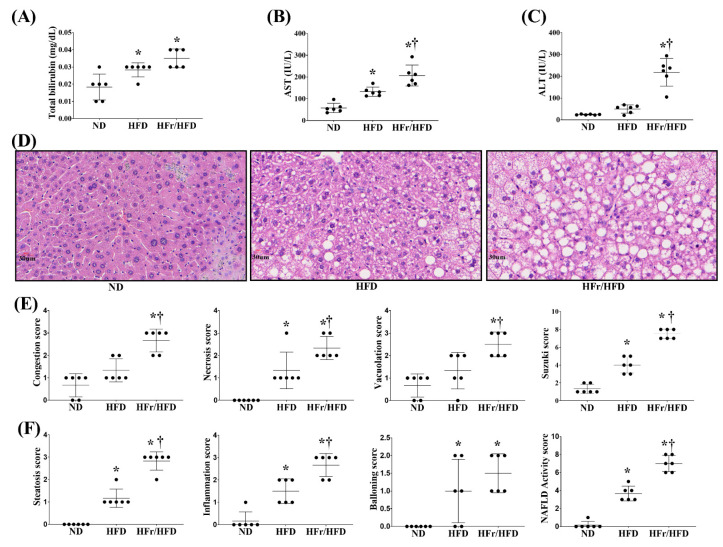
Plasma concentrations of (**A**) alanine aminotransferase (ALT), (**B**) alanine aspartate (AST), and (**C**) total bilirubin. (**D**) Representative microscopic findings of liver stained with hematoxylin–eosin (×200) and (**E**) parameters of Suzuki score (congestion, necrosis and vacuolation). (**F**) Parameters of NAFLD activity score (steatosis, inflammation, and ballooning degeneration). Data were obtained from 6 mice in each group and presented as mean ± standard deviation. ND: normal diet group. HFD: high-fat diet group. HFr/HFD: high-fat diet plus 30% fructose in drinking water group. NAFLD: non-alcoholic fatty liver disease. * *p* < 0.05, versus the ND group; † *p* < 0.05, the HFr/HFD group versus the HFD group.

Figure 3 illustrates the plasma concentrations of hepatic enzymes and the liver histological characteristics of the ND, HFD, and HFr/HFD groups. The plasma concentrations of aspartate aminotransferase (AST) (Figure 3B) and total bilirubin (Figure 3C) in the HFD group were both significantly higher than those in the ND group (*p* = 0.003 and = 0.03, respectively), whereas alanine aminotransferase (ALT) (Figure 3A) in the HFD group was not significantly different from that in the ND group (*p* = 0.5). The plasma concentrations of ALT, AST, and total bilirubin in the HFr/HFD group were all significantly higher than those in the ND group (all *p* < 0.001). Moreover, the plasma concentrations of ALT and AST in the HFr/HFD group were both significantly higher than those in the HFD group (*p* < 0.001 and =0.004, respectively). Plasma concentrations of total bilirubin in the HFr/HFD and the HFD groups were not significantly different (*p* = 0.15).

Figure 3D represents the histological characteristics of liver in each test group, using hematoxylin and eosin staining. Comparing to the ND group, significant liver injury and NAFLD characteristics in the HFD and HFr/HFD groups were observed. Figure 3E represents the individual parameter scores and the liver injury scores (i.e., the sums of the individual parameter scores) of each test group. The scores of congestion and vacuolation in the HFr/HFD group were both significantly higher than those in the ND and HFD groups (congestion: *p* < 0.001 and =0.001, respectively; vacuolation: *p* < 0.001 and =0.010, respectively); whereas the scores of congestion and vacuolation in the HFD and ND groups were not significantly different (*p* = 0.09 and =0.20, respectively). Notably, the necrosis scores in the HFD and HFr/HFD groups were both significantly higher than that in the ND group (*p* = 0.002 and <0.001, respectively). Moreover, the necrosis score in the HFr/HFD group was significantly higher than that in the HFD group (*p* = 0.01). The liver injury scores in the HFD and HFr/HFD groups were both significantly higher than that in the ND group (both *p* < 0.001). Moreover, the liver injury score in the HFr/HFD group was significantly higher than that in the HFD group (*p* < 0.001).

Figure 3F represents the individual parameter scores and the NAFLD activity score (i.e., the sums of the individual parameter scores) in each test group. The scores of steatosis and inflammation in the HFD and HFr/HFD groups were both significantly higher than those in the ND groups (steatosis: both *p* < 0.001; inflammation: both *p* < 0.001). Moreover, the scores of steatosis and inflammation in the HFr/HFD group were both significantly higher than those in the HFD group (*p* < 0.001 and =0.003, respectively). The ballooning degeneration score in the HFD and HFr/HFD groups were also significantly higher than that in the ND group (*p* = 0.030 and <0.001, respectively); whereas the ballooning degeneration scores in the HFD and HFr/HFD groups were not significantly different, either (*p* = 0.35). The NAFLD activity scores in the HFD and HFr/HFD groups were both significantly higher than that in the ND group (both *p* < 0.001). Moreover, the NAFLD activity score in the HFr/HFD group was significantly higher than that in the HFD group (*p* < 0.001).

### 3.4. Total Proteome Alteration

**Figure 4:**
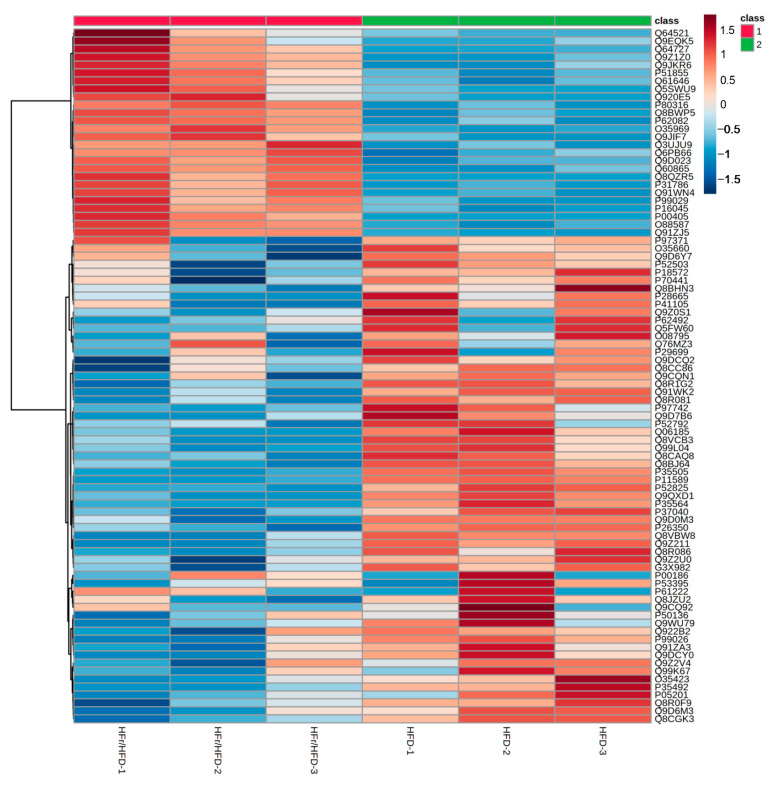
Heatmap of proteins identified in liver tissues from mice of the HFD and HFr/HFD groups. Data were obtained from 3 mice in each group. The intensities of proteins were processed with log10 transformation and presented in colors, ranging from red to blue. Red indicates protein upregulation and blue indicates protein downregulation in the HFr/HFD group, compared with the HFD group. HFD: high-fat diet group. HFr/HFD: high-fat diet plus 30% fructose in drinking water group.

A label-free LC-MS/MS analysis was conducted to determine the total proteome alteration in mouse liver between the HFD and the HFr/HFD groups. Three biological replicates were performed for each of the HFD and HFr/HFD groups. Our analysis identified 1075 proteins with expression alterations between the HFD and the HFr/HFD groups. Figure 4 illustrates the heatmap.

### 3.5. Comparison of Canonical Pathways

**Figure 5:**
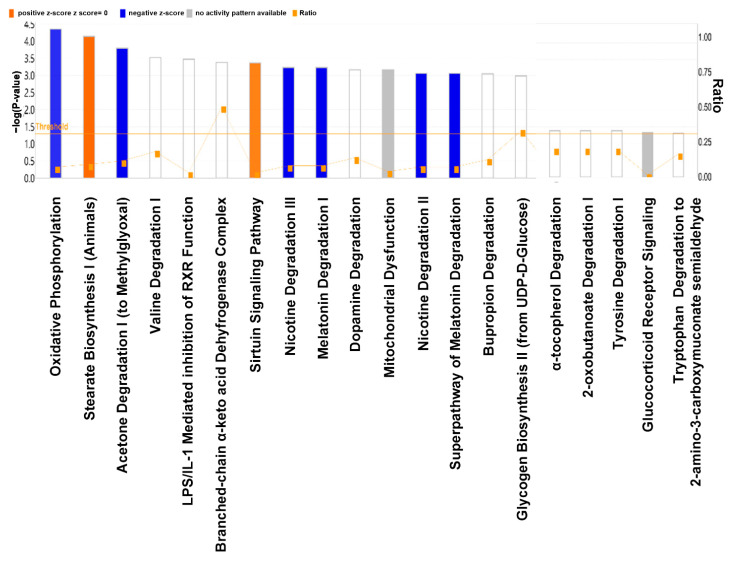
A comparison of the canonical pathways in the liver between the HFD and HFr/HFD groups using Ingenuity Pathway Analysis. An analysis of the pathway is depicted horizontally, and the unadjusted *p*-value is shown vertically (−log(*p*)). Expressions of canonical pathways are considered to be significantly different between these two groups at a *p* ≤ 0.05, with the threshold set to 1.3. Orange indicates pathway upregulation and blue indicates pathway downregulation in the HFr/HFD group, compared with the HFD group. A gray bar indicates that there can be no prediction. HFD: high-fat diet group. HFr/HFD: high-fat diet plus 30% fructose in drinking water group.

Figure 5 illustrates the IPA data depicting the comparison of the canonical pathways in the liver between the HFD and HFr/HFD groups. Our data demonstrated that, in comparison with the HFD group, the hepatic mitochondrial oxidative phosphorylation (OXPHOS) pathway was the most significantly downregulated canonical pathway in the HFr/HFD group.

### 3.6. Dysregulated Proteins in the Mitochondrial OXPHOS Pathway

**Figure 6:**
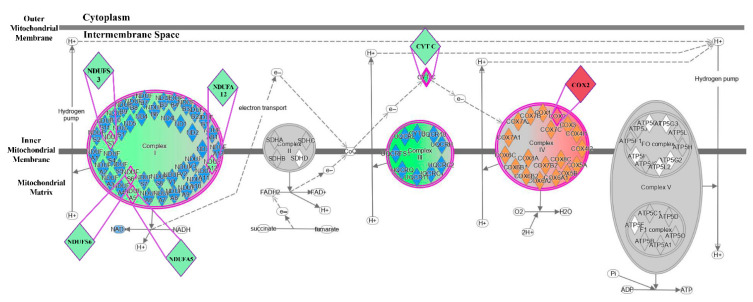
The differences between the HFD and the HFr/HFD groups in pathway and protein expression associated with the respiratory chain in liver, analyzed using the Ingenuity Pathway Analysis. Purple denotes pathways that are upregulated in the HFr/HFD group, compared with the HFD group. Green indicates pathway and protein downregulation in the HFr/HFD group, compared with the HFD group. Red indicates pathway and protein upregulation in the HFr/HFD group, compared with the HFD group. A gray background indicates pathway and protein unaffected. HFD: high-fat diet group. HFr/HFD: high-fat diet plus 30% fructose in drinking water group.

As stated above, the mitochondrial OXPHOS pathway was the most downregulated canonical pathway in the HFr/HFD group when compared with the HFD group. We further analyzed the dysregulated proteins involved in OXPHOS complexes using IPA (Figure 6). Our data demonstrated more downregulation of OXPHOS complexes I and III, and more upregulation of OXPHOS complex IV in the HFr/HFD group, when compared with the HFD group. Furthermore, the proteins of nicotinamide adenine dinucleotide (NAD) + hydrogen (H) (NADH): ubiquinone oxidoreductase subunits S3 (NDUFS3), S6 (NDUFS6), A5 (NDUFA5), and A12 (NDUFA12) in OXPHOS complex I, and cytochrome C (CYT C) in OXPHOS complex III were downregulated in the HFr/HFD group, when compared with the HFD group. In contrast, cytochrome C oxidase subunit 2 (Cox2) in OXPHOS complex IV was upregulated in the in the HFr/HFD group when compared with the HFD group. We noted that expression of OXHPOS complexes II and V were not significantly different between the HFD and HFr/HFD groups.

### 3.7. Immunoblotting Assay

**Figure 7:**
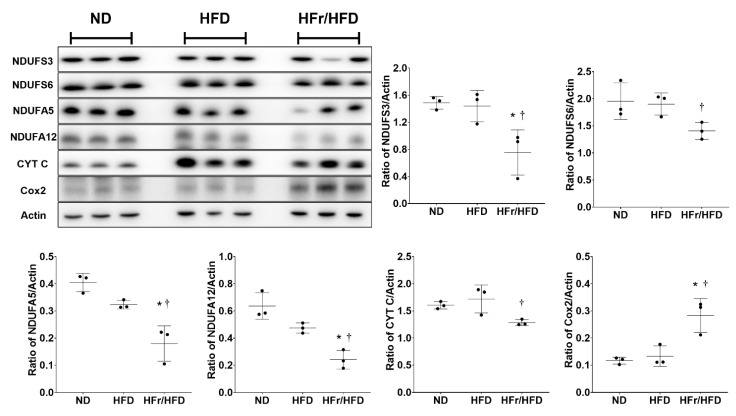
Representative gel photography of immunoblotting assay in liver tissues for the levels of NDUFS3, NDUFS6, NDUFA5, NDUFA12, CYT C, Cox2, and Actin (the internal standard) in the ND, HFD and HFr/HFD groups. Quantification of band intensity represented as ratio to targeting protein over Actin. Data were derived from three mice in each group and presented as mean ± standard deviation. NDUFS3: NADH: ubiquinone oxidoreductase subunits S3, NDUFS6: NADH: ubiquinone oxidoreductase subunits S6, NDUFA5: NADH: ubiquinone oxidoreductase subunits A5, and NDUFA12: NADH: ubiquinone oxidoreductase subunits A12. CYT C: cytochrome C. Cox2: cytochrome C oxidase subunit 2. ND: normal diet group. HFD: high-fat diet group. HFr/HFD: high-fat diet plus 30% fructose in drinking water group. * *p* < 0.05, versus the ND group; † *p* < 0.05, the HFr/HFD group versus the HFD group. Note. The original images of immunoblotting assay can be found in the Supplemental Materials: Figure S1.

To validate the IPA data described above, we performed immunoblotting assays to analyze the relative levels of the dysregulated liver proteins involved in the mitochondrial OXPHOS pathway. Figure 7 illustrates the immunoblotting assay data and the expression levels of NDUFS3, NDUFS6 NDUFA5, NDUFA12, CYT C and Cox2 in each test group. Our data demonstrated that the expression levels of NDUFS3, NDUFS6, NDUFA5, NDUFA12, CYT C, and Cox2 in the HFD and ND groups were not significantly different (*p* = 0.97, =0.96, =0.13, =0.08, =0.65, and =0.89, respectively). Our data also demonstrated that the expression levels of NDUFS3, NDUFA5, and NDUFA12 in the HFr/HFD group were significantly lower than those in the ND group (*p* = 0.023, =0.002, and =0.003, respectively). In contrast, the expression level of Cox2 in the HFr/HFD group was significantly higher than that in the ND group (*p* = 0.007). Moreover, the expression levels of NDUFS6 and CYT C in the HFr/HFD and ND groups were not significantly different (*p* = 0.08 and =0.10, respectively). Our data further demonstrated that the expression levels of NDUFS3, NDUFS6, NDUFA5, NDUFA12, and CYT C in the HFr/HFD were significantly lower than those in the HFD group (*p* < 0.001, =0.03, <0.001, <0.001 and <0.001, respectively). Notably, the expression level of Cox2 was significantly higher in the HFr/HFD group than in the HFD group (*p* = 0.002).

## 4. Discussion

Our data reaffirm that long-term feeding of either HFr/HFD or HFD induces obesity, metabolic derangements, liver injury, and NAFLD in mice, and is consistent with previous findings in the literature [5]. Our data further reaffirm the different impacts that HFr/HFD and HFD may have on inducing liver injury and NAFLD progression in mice, namely, long-term feeding of HFr/HFD induces more severe liver injury and more advanced NAFLD than long-term feeding of HFD alone [5,10]. We suggest that long-term HFr/HFD feeding should be the preferred animal model in the study of NAFLD, over HFD alone.

The different effects of HFr/HFD and HFD on inducing liver injury and NAFLD progression are well established. Previous data indicated that excessive fructose intake and excessive fatty acids intake can exert similar effects on inducing lipolysis and de novo lipogenesis alterations, fatty acid oxidation impairment, endoplasmic reticulum stress, inflammation in liver, and subsequently liver injury and NAFLD [26,27,28]. Based on these data, one would speculate that simultaneously consuming excessive fructose and fatty acids (such as HFr/HFD) may thus exert more adverse impacts than consuming excessive fatty acids alone (such as HFD). In line with this notion, it is thus reasonable to observe that long-term HFr/HFD feeding induces more severe liver injury and more advanced NAFLD than does long-term HFD feeding in mice. These data provide evidence to support the concept that fructose may aggravate the adverse effects of fatty acids and thus exert more adverse impacts than fatty acid alone. However, as mentioned above, the molecular mechanism(s) remains to be elucidated.

This study was thus conducted to elucidate the molecular mechanisms underlying the different effects of HFr/HFD and HFD on inducing liver injury and NAFLD progression using a proteomics approach. Based on our analysis of liver tissues, we found that there were differentially expressed proteins between the groups with HFr/HFD and HFD. An analysis of bioinformatics data revealed that these proteins played a prominent role in a wide range of metabolic processes. Among them, the mitochondrial OXPHOS pathway was identified as the most significantly downregulated pathway in the HFr/HFD mice, compared with the HFD mice. The mitochondrial OXPHOS pathway comprises five complexes, namely complex I–V [29]. The complex I (also known as NADH dehydrogenase) catalyzes oxidation of NADH [30]. The complex II (also known as succinate dehydrogenase) oxidizes succinate and reduces ubiquinone [31]. The complex III (also known as Q-cytochrome c oxidoreductase) oxidizes ubiquinol and reduces cytochrome c [32]. The complex IV (also known as cytochrome c oxidase) mediates electron transfer to oxygen and hydrogen [33]. The complex V (also known as adenosine triphosphate [ATP] synthase) catalyzes ATP formation using adenosine diphosphate [34]. Previous data demonstrated that patients with NAFLD have decreased respiratory chain activity by 37% in complex I, 42% in complex II, 30% in complex III, 38% in complex IV, and 58% in complex V compared with those without NAFLD [35]. Of note, among the five complexes, our bioinformatics data further identified downregulations of complexes I and III, and upregulation of complex IV in the HFr/HFD mice, comparing with the HFD mice, whereas expression of complexes II and V were not significantly different between these two groups. Moreover, our bioinformatics data with immunoblotting assay validation identified that, among the convoluted components of complex I, four NADH: ubiquinone oxidoreductase subunits proteins (namely NDUFS3, NDUFS6, NDUFA5, and NDUFA12) were downregulated in the HFr/HFD mice comparing with the HFD mice. Our data also identified that CYT C in complex III was downregulated in the HFr/HFD mice, comparing with the HFD mice. Moreover, upregulation of Cox2 in complex IV was noted in the HFr/HFD mice comparing with the HFD mice. These data provide clear evidence for the first time to highlight that the molecular mechanism underlying the different effects of HFr/HFD and HFD on inducing liver injury in NFLFD mice should involve the mitochondrial OXPHOS pathway, especially complexes I, III, IV, and the specific proteins of NADH: ubiquinone oxidoreductase subunits, CYT C, and Cox2. The above-mentioned pathway and specific proteins can thus be the potential therapeutic targets. Based on these data, novel therapy against NAFLD may very likely be developed in the future.

The mitochondrial OXPHOS pathway (also known as the electron transport chain or mitochondrial respiratory chains that locate in the mitochondrial inner membrane) is the machinery for ATP production [36]. Downregulation (or impairment) of the mitochondrial OXPHOS pathway is considered as an indicator of mitochondrial dysfunction [29,36]. Notably, the crucial role of hepatic mitochondrial dysfunction in mediating the development of NAFLD has long been established [35,37]. Data from the present study are thus consistent with previous results [35,37]. Collectively, these data indicate that the different effects of HFr/HFD and HFD on inducing liver injury in NAFLD mice should involve their effects on causing mitochondrial dysfunction. It has been demonstrated that hepatic mitochondrial dysfunction may act through inducing alterations of lipid homeostasis, increases in ROS generation and lipid peroxidation, upregulation of cytokines, and apoptosis in the liver to cause NAFLD [35,37,38]. The main mechanisms that mediate the development of mitochondrial dysfunction in NAFLD involve obesity-induced inflammatory response and oxidative stress [35,39] as well as obesity-induced excessive carbohydrate and fatty acid supply to mitochondria, a process that may overwhelm the tricarboxylic acid cycle and the mitochondrial respiratory chain (namely the OXPHOS pathway) [40]. Notably, fructose can exert adverse impacts and cause mitochondrial dysfunction that are similar to fatty acids [27,28]. Inclusion of fructose in high-fat diet (namely HFr/HFD) may thus aggravate the adverse effects of HFD and cause more severe mitochondrial dysfunction than HFD alone. This concept is supported by data from the present study.

It is evident from these results that proteomics can be an effective method to provide high-throughput information on the complex pathogenesis of NAFLD. Our data, in concert with previous results [10], support the concept that mitochondrial dysfunction, especially the mitochondrial OXPHOS pathway, plays a crucial role in mediating the different impacts of HFr/HFD and HFD on inducing liver injury in the development and progression of NAFLD. However, the mechanisms that mediate the effects of HFr/HFD on downregulating the mitochondrial OXPHOS pathway, comparing with HFD, in mice remain to be elucidated. Previous data indicated that NAFLD-induced mitochondrial impairment is associated with increased degradation of OXPHOS subunits mediated by mitophagy [41]. In line with this concept, we therefore conjecture that the same mechanism may also participate in mediating the different impacts of HFr/HFD and HFD observed in the present study. More studies are needed before further conclusion can be drawn. 

NAFLD is frequently associated with elevated fasting plasma triglyceride [4,42]. The mechanisms involve insulin resistance, altered lipoprotein metabolism, and increased hepatic synthesis and secretion of VLDL, i.e., the carrier of triglyceride to adipose tissues and muscles [4,42]. However, NAFLD may progress from simple steatosis to steatohepatitis, fibrosis and cirrhosis [5]. With hepatocellular injury and dysfunction associated with advanced NAFLD, lipoprotein metabolism will be impaired and hepatic syntheses of VLDL and triglyceride as well as their exports to tissues will be decreased [43]. This phenomenon is observed in this study, as our data demonstrated that the fasting plasma triglyceride and VLDL levels in the HFD group were not significantly different from those in the ND group, though a trend of higher fasting plasma triglyceride and VLDL levels in the HFD group was observed. Moreover, with NAFLD progression to liver fibrosis and cirrhosis, clinical data demonstrated that a low fasting plasma triglyceride (rather than a high fasting plasma triglyceride) can be observed [43,44]. Based on these findings, low fasting plasma triglyceride is now considered as a non-invasive marker of advanced NAFLD (especially, liver fibrosis and cirrhosis) [44]. As mentioned above, data from this study, in concert with those from previous studies [5,10], confirmed that the long-term feeding of HFr/HFD induces more severe liver injury and NAFLD progression than the long-term feeding of HFD alone in mice. Previous data further demonstrated that mice with long-term HFr/HFD feeding had lower hepatic synthesis of triglyceride, compared with mice with long-term HFD feeding alone [45]. In line with this concept, it is thus reasonable to observe our data that fasting plasma triglyceride level in the HFr/HFD group was lower than those in the HFD and ND groups. Of note, although our data demonstrated no significant difference in plasma VLDL concentrations between the HFr/HFD and ND groups, we did observe a trend of lower plasma VLDL concentration in the HFr/HFD group. Previous data indicated that fasting plasma triglyceride can serve as a proxy of VLDL, as expression of fasting plasma triglyceride is tightly associated with VLDL [4,42]. Our data seem to support this concept.

It is important to note that this study has several limitations. For instance, the animal models cannot represent all NAFLD situations, as injuries caused by various degrees and durations of NAFLD may vary in severity. Moreover, this study only evaluated the outcomes of NAFLD after 12 weeks of HFr/HFD and HFD feedings. The results of a longer follow-up course may, however, provide additional insight. As liver disease progresses (such as non-alcoholic steatohepatitis, fibrosis, and cirrhosis), further studies are necessary to evaluate the impacts of the mitochondrial OXPHOS pathway and the other relevant mechanisms may have in this regard. In addition, though this study highlights the crucial role of the mitochondrial OXPHOS pathway in this regard, it remains unclear whether preserving mitochondrial respiratory function of the liver can exert therapeutic effects against NAFLD-induced chronic liver disease. To draw further conclusions, more research is needed.

## 5. Conclusions

Long-term high-fructose/high-fat diet feeding downregulates the hepatic mitochondrial oxidative phosphorylation pathway in mice, comparing with long-term high-fat diet feeding.

## Data Availability

Data is contained within the article and supplementary material.

## Supplementary Materials

The following supporting information can be downloaded at: https://www.mdpi.com/article/10.3390/cells11213425/s1, Figure S1: original images of immunoblotting assay.

## References

[1] Kawano Y., Cohen D.E. (2013). Mechanisms of hepatic triglyceride accumulation in non-alcoholic fatty liver disease. J. Gastroenterol..

[2] Riazi K., Azhari H., Charette J.H., Underwood F.E., King J.A., Afshar E.E., Swain M.G., Congly S.E., Kaplan G.G., Shaheen A.A. (2022). The prevalence and incidence of NAFLD worldwide: A systematic review and meta-analysis. Lancet Gastroenterol. Hepatol..

[3] Rector R.S., Thyfault J.P., Uptergrove G.M., Morris E.M., Naples S.P., Borengasser S.J., Mikus C.R., Laye M.J., Laughlin M.H., Booth F.W. (2010). Mitochondrial dysfunction precedes insulin resistance and hepatic steatosis and contributes to the natural history of non-alcoholic fatty liver disease in an obese rodent model. J. Hepatol..

[4] Wierzbicki A.S., Oben J. (2012). Nonalcoholic fatty liver disease and lipids. Curr. Opin. Lipidol..

[5] Cohen J.C., Horton J.D., Hobbs H.H. (2011). Human fatty liver disease: Old questions and new insights. Science.

[6] Hill J.O., Lin D., Yakubu F., Peters J.C. (1992). Development of dietary obesity in rats: Influence of amount and composition of dietary fat. J. Int. Assoc. Study Obes..

[7] Romieu I., Willett W.C., Stampfer M.J., Colditz G.A., Sampson L., Rosner B., Hennekens C.H., Speizer F.E. (1988). Energy intake and other determinants of relative weight. Am. J. Clin. Nutr..

[8] Sanches S.C., Ramalho L.N., Augusto M.J., da Silva D.M., Ramalho F.S. (2015). Nonalcoholic steatohepatitis: A search for factual animal models. BioMed Res. Int..

[9] Ito M., Suzuki J., Tsujioka S., Sasaki M., Gomori A., Shirakura T., Hirose H., Ito M., Ishihara A., Iwaasa H. (2007). Longitudinal analysis of murine steatohepatitis model induced by chronic exposure to high-fat diet. Hepatol. Res..

[10] García-Berumen C.I., Ortiz-Avila O., Vargas-Vargas M.A., Del Rosario-Tamayo B.A., Guajardo-López C., Saavedra-Molina A., Rodríguez-Orozco A.R., Cortés-Rojo C. (2019). The severity of rat liver injury by fructose and high fat depends on the degree of respiratory dysfunction and oxidative stress induced in mitochondria. Lipids Health Dis..

[11] Machado M.V., Michelotti G.A., Xie G., Almeida Pereira T., Boursier J., Bohnic B., Guy C.D., Diehl A.M. (2015). Mouse models of diet-induced nonalcoholic steatohepatitis reproduce the heterogeneity of the human disease. PLoS ONE.

[12] Stocks B., Gonzalez-Franquesa A., Borg M.L., Björnholm M., Niu L., Zierath J.R., Deshmukh A.S. (2022). Integrated Liver and Plasma Proteomics in Obese Mice Reveals Complex Metabolic Regulation. Mol. Cell. Proteom..

[13] Malik A.N., Simões I.C.M., Rosa H.S., Khan S., Karkucinska-Wieckowska A., Wieckowski M.R. (2019). A diet induced maladaptive increase in hepatic mitochondrial DNA precedes OXPHOS defects and may contribute to non-alcoholic fatty liver disease. Cells.

[14] Woszczynski M., Ledwon J., Dabrowska M., Dadlez M., Ostrowski J. (2014). Mitochondrial-related proteomic changes during obesity and fasting in mice are greater in the liver than skeletal muscles. Funct. Integr. Genom..

[15] Sabidó E., Wu Y., Bautista L., Porstmann T., Chang C.Y., Vitek O., Stoffel M., Aebersold R. (2013). Targeted proteomics reveals strain-specific changes in the mouse insulin and central metabolic pathways after a sustained high-fat diet. Mol. Syst. Biol..

[16] Zembroski A.S., Buhman K.K., Aryal U.K. (2021). Proteome and phosphoproteome characterization of liver in the postprandial state from diet-induced obese and lean mice. J. Proteom..

[17] Chiang M.D., Chang C.Y., Shih H.J., Le V.L., Huang Y.H., Huang C.J. (2022). Exosomes from Human Placenta Choriodecidual Membrane-Derived Mesenchymal Stem Cells Mitigate Endoplasmic Reticulum Stress, Inflammation, and Lung Injury in Lipopolysaccharide-Treated Obese Mice. Antioxidants.

[18] Chyau C.C., Wang H.F., Zhang W.J., Chen C.C., Huang S.H., Chang C.C., Peng R.Y. (2020). Antrodan Alleviates High-Fat and High-Fructose Diet-Induced Fatty Liver Disease in C57BL/6 Mice Model via AMPK/Sirt1/SREBP-1c/PPARγ Pathway. Int. J. Mol. Sci..

[19] Kohli R., Kirby M., Xanthakos S.A., Softic S., Feldstein A.E., Saxena V., Tang P.H., Miles L., Miles M.V., Balistreri W.F. (2010). High-fructose, medium chain trans fat diet induces liver fibrosis and elevates plasma coenzyme Q9 in a novel murine model of obesity and nonalcoholic steatohepatitis. Hepatology.

[20] Lee J.S., Jun D.W., Kim E.K., Jeon H.J., Nam H.H., Saeed W.K. (2015). Histologic and metabolic derangement in high-fat, high-fructose, and combination diet animal models. Sci. World J..

[21] Aslam M.N., Bassis C.M., Zhang L., Zaidi S., Varani J., Bergin I.L. (2016). Calcium Reduces Liver Injury in Mice on a High-Fat Diet, Alterations in Microbial and Bile Acid Profiles. PLoS ONE.

[22] Hjelkrem M., Stauch C., Shaw J., Harrison S.A. (2011). Validation of the non-alcoholic fatty liver disease activity score. Aliment. Pharm. Ther..

[23] Shih H.J., Chang C.Y., Huang I.T., Tsai P.S., Han C.L., Huang C.J. (2021). Testicular torsion–detorsion causes dysfunction of mitochondrial oxidative phosphorylation. Andrology.

[24] Liu H., Sadygov R.G., Yates J.R. (2004). A model for random sampling and estimation of relative protein abundance in shotgun proteomics. Anal. Chem..

[25] Chang C.Y., Chen K.Y., Shih H.J., Chiang M., Huang I.T., Huang Y.H., Huang C.J. (2021). Let-7i-5p mediates the therapeutic effects of exosomes from human placenta choriodecidual membrane-derived mesenchymal stem cells on mitigating endotoxin-induced mortality and liver injury in high-fat diet-induced obese mice. Pharmaceuticals.

[26] Khan R.S., Bril F., Cusi K., Newsome P.N. (2019). Modulation of insulin resistance in nonalcoholic fatty liver disease. Hepatology.

[27] Francey C., Cros J., Rosset R., Crézé C., Rey V., Stefanoni N., Schneiter P., Tappy L., Seyssel K. (2019). The extra-splanchnic fructose escape after ingestion of a fructose–glucose drink: An exploratory study in healthy humans using a dual fructose isotope method. Clin. Nutr. ESPEN.

[28] Softic S., Stanhope K.L., Boucher J., Divanovic S., Lanaspa M.A., Johnson R.J., Kahn C.R. (2020). Fructose and hepatic insulin resistance. Crit. Rev. Clin. Lab. Sci..

[29] Guo R., Zong S., Wu M., Gu J., Yang M. (2017). Architecture of human mitochondrial respiratory megacomplex I_2_III_2_IV_2_. Cell.

[30] Hirst J. (2005). Energy transduction by respiratory complex I–an evaluation of current knowledge. Biochem. Soc. Trans..

[31] Cecchini G. (2003). Function and structure of complex II of the respiratory chain. Annu Rev Biochem..

[32] Crofts A.R. (2004). The cytochrome bc 1 complex: Function in the context of structure. Annu. Rev. Physiol..

[33] Calhoun M.W., Thomas J.W., Gennis R.B. (1994). The cytochrome oxidase superfamily of redox-driven proton pumps. Trends Biochem. Sci..

[34] Junge W., Nelson N. (2015). ATP synthase. Annu. Rev. Biochem..

[35] Pérez-Carreras M., Del Hoyo P., Martín M.A., Rubio J.C., Martín A., Castellano G., Colina F., Arenas J., Solis-Herruzo J.A. (2003). Defective hepatic mitochondrial respiratory chain in patients with nonalcoholic steatohepatitis. Hepatology.

[36] Mitchell P. (1961). Coupling of phosphorylation to electron and hydrogen transfer by a chemi-osmotic type of mechanism. Nature.

[37] Bohinc B.N., Diehl A.M. (2012). Mechanisms of disease progression in NASH: New paradigms. Clin. Liver Dis..

[38] Begriche K., Igoudjil A., Pessayre D., Fromenty B. (2006). Mitochondrial dysfunction in NASH: Causes, consequences and possible means to prevent it. Mitochondrion.

[39] Sunny N.E., Parks E.J., Browning J.D., Burgess S.C. (2011). Excessive hepatic mitochondrial TCA cycle and gluconeogenesis in humans with nonalcoholic fatty liver disease. Cell Metab..

[40] De Mello A.H., Costa A.B., Engel J.D.G., Rezin G.T. (2018). Mitochondrial dysfunction in obesity. Life Sci..

[41] Lee K., Haddad A., Osme A., Kim C., Borzou A., Ilchenko S., Allende D., Dasarathy S., McCullough A., Sadygov R.G. (2018). Hepatic mitochondrial defects in a nonalcoholic fatty liver disease mouse model are associated with increased degradation of oxidative phosphorylation subunits. Mol. Cell. Proteom..

[42] Fabbrini E., Sullivan S., Klein S. (2010). Obesity and nonalcoholic fatty liver disease: Biochemical, metabolic, and clinical implications. Hepatology.

[43] Cicognani C., Malavolti M., Morselli-Labate A.M., Zamboni L., Sama C., Barbara L. (1997). Serum lipid and lipoprotein patterns in patients with liver cirrhosis and chronic active hepatitis. Arch. Intern. Med..

[44] Jiang Z.G., Tsugawa Y., Tapper E.B., Lai M., Afdhal N., Robson S.C., Mukamal K.J. (2015). Low-fasting triglyceride levels are associated with non-invasive markers of advanced liver fibrosis among adults in the United States. Aliment. Pharmacol. Ther..

[45] Siersbæk M.S., Ditzel N., Hejbøl E.K., Præstholm S.M., Markussen L.K., Avolio F., Li L., Lehtonen L., Hansen A.K., Schrøder H.D. (2020). C57BL/6J substrain differences in response to high-fat diet intervention. Sci. Rep..

