# Supplementary figures and images for: High-Fructose/High-Fat Diet Downregulates the Hepatic Mitochondrial Oxidative Phosphorylation Pathway in Mice Compared with High-Fat Diet Alone
Source: Cells. 2022 Oct 29;11(21):3425. doi: 10.3390/cells11213425 (PMC9656843; doi:10.3390/cells11213425)

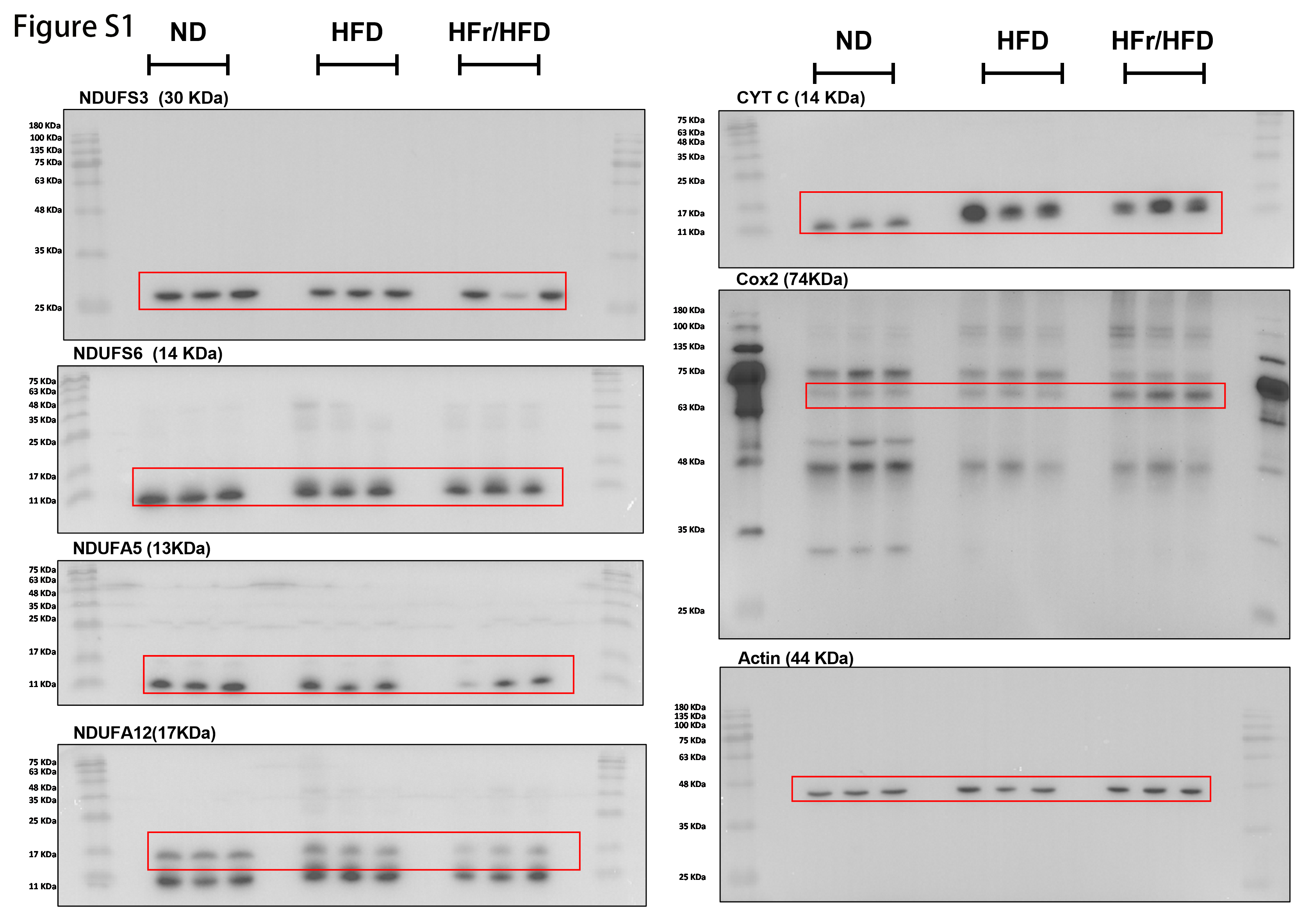

Supplement: Supplementary file 1 [file cells-11-03425-s001.zip › cells-1953815-supplementary.tif]
